# Supplementary material for: A scoping review key elements and effects of cardiovascular disease management programs based on community-based participatory research
Source: PLoS One. 2023 Jan 20;18(1):e0279563. doi: 10.1371/journal.pone.0279563 (PMC9858102; doi:10.1371/journal.pone.0279563)
Supplement: Attachment — Submitted filename: authors_response_0602.docx [file pone.0279563.s003.docx]

Authors’ Response for the Reviewers’ Report:

\# Editor

Thank you for your good comments on this study. You can check the revised contents through the track change.

1. Literature search results are "results" not methods. The flow diagram and literature search results/numbers should be in the first paragraph of results, not methods.

🡪 Generally, in scoping reviews and systematic reviews, the study selection process is described in the research method.

2. Please ensure that your manuscript meets PLOS ONE's style requirements, including those for file naming. 
🡪 After reviewing *PLOS ONE’*s style requirements, we revised the manuscript and references.

3. During the internal evaluation of your manuscript we have noted that the literature search was completed in April 2020. Please consider whether an update to the literature search should be conducted since scoping reviews should provide an up-to-date overview of the research topic covered.
🡪 After searching for additional published studies from April 2020 to April 2022, the results were added and described.

4. In your Data Availability statement, you have not specified where the minimal data set underlying the results described in your manuscript can be found. PLOS defines a study's minimal data set as the underlying data used to reach the conclusions drawn in the manuscript and any additional data required to replicate the reported study findings in their entirety.

🡪 In this study, the data extraction process was presented in detail in the research method. Tables 2–5 present the result of the study, that is, the final data. The search strategies were attached as supplementary data.

5. Your abstract cannot contain citations. Please only include citations in the body text of the manuscript, and ensure that they remain in ascending numerical order on first mention.
🡪 The abstract does not contain any citations any longer.

6. Please include captions for your Supporting Information files at the end of your manuscript, and update any in-text citations to match accordingly.

🡪 We attached the search strategies for each database and PRISM-ScR at the end of our manuscript.

7. Please review your reference list to ensure that it is complete and correct. 
🡪 We reviewed and revised the reference list.

Reviewer #1:

Thank you for your good comments on this study. You can check the revised contents through the track change.

Thanks for your efforts regarding this well written review summarizing current CBPR practices, the research question is an important one in the current Cardiology community practice which was properly addressed by the authors, also results led to proper conclusions
🡪 Thank you for your encouraging comments. We have made revisions based on your feedback, which, we believe, has improved the quality of our paper.

1. Abstract: Any reason for choosing the age above 18 to be enrolled in the review?
🡪 The target population of this study is adults. Many studies define people over the age of 18 as adults, and thus we applied that criterion in this study.

2. Abstract: It was written in the abstract in all 6 RCTs without any brief explanation of them in previous paragraph.
🡪 We added a brief explanation of this point, as follows.

***Page 2 Line 35 to 37***

In addition, among the 21 studies selected were 6 RCTs, all of which showed a significant positive effect in experimental groups compared to control groups.

3. Materials and methods: Describe review method developed by Arcsey and O'Malley.
🡪 The review method developed by Arcsey and O’Malley is described in lines 96–100, on page 6.

4. Describe PRISMA extension for scoping review check list.
🡪 PRISMA-SCR is an abbreviation of ‘Preferred Reporting Items for Systematic Reviews and Meta-Analyses Extension for Scoping Reviews’, and the full term is presented inside parentheses.

***Page 5 Lines 98 to 99:*** In the process of conducting this scoping review and reporting the results, we abided by the PRISMA-ScR (Preferred Reporting Items for Systematic Reviews and Meta-Analyses Extension for Scoping Reviews) checklist [22].

5. Please explain search terms Cardiovasc* , vascul* disease, PAR ? , participat ?
🡪 * is a symbol that has the function to search for all words that match the spelling located before * when searching in electronic databases such as PubMed and CINAHL (Searching using 'Cardiovasc*' searches for 'Cardiovascular', 'Cardiovasculitis', etc). PAR, which is abbreviation for Participatory Action Research, was selected in the search because it is a research form similar to CBPR. ‘participat*’ was revised to ‘participant*’ because it was a typo.

6. In line 270, 271 describe each model

🡪 ‘Intervention mapping’ and ‘precede–proceed model’ are the names of the health planning models. Since these models were used in the selected studies, we simply listed the names of models in the manuscripts. We deleted 'CATCH-PATH model' as it was confirmed that it was not a planning model.

7. Details of statistics of your chosen studies need to be properly highlighted.

🡪 The statistical methods used in the selected study were added to Table 5.

8. Rewrite both tables 2 and 3 (establishing partnership, CBPR principle) in a simple fashion.
🡪 The text of Tables 2 and 3 has been restated more concisely. Lines 227–247.

Reviewer #2:

Thank you for your good comments on this study. You can check the revised contents through the track change.

The authors presented a scoping review about key elements and effects of cardiovascular disease management programs based on community-based participatory research. The idea is interesting, the article is well written. I thank the authors for their effort
🡪 Thank you for your encouraging comments. We have made revisions based on your feedback, which, we believe, has improved the quality of our paper.

1. In table 1, there is a study where adults & children were included and another one that did not report the included age. This does not meet the criteria of your selection (>18 y).
🡪 Although Schroeder et al. (2017) studied both adults and children, we included results from the study with adults because we were able to confirm them. We deleted “children” from the table because it could make confusion for readers.

2. The introduction and the discussion need to be shortened.

🡪 We reviewed the introduction and discussion parts and described them more concisely. We deleted the description of why we used the scoping review method at the beginning of the discussion.

3. The answers for the research questions should be clarified during the discussion.

🡪 The discussion was revised to focus on the answers to the research questions.
