# Supplementary material for: A scoping review key elements and effects of cardiovascular disease management programs based on community-based participatory research
Source: PLoS One. 2023 Jan 20;18(1):e0279563. doi: 10.1371/journal.pone.0279563 (PMC9858102; doi:10.1371/journal.pone.0279563)
Supplement: Attachment — Submitted filename: authors_response_221106.docx [file pone.0279563.s004.docx]

Authors’ Response for the Reviewers’ Report:

\# Editor

Thank you for your good comments on this study. We marked the modified part in red.

1. Please review your reference list to ensure that it is complete and correct. If you have cited papers that have been retracted, please include the rationale for doing so in the manuscript text, or remove these references and replace them with relevant current references. Any changes to the reference list should be mentioned in the rebuttal letter that accompanies your revised manuscript. If you need to cite a retracted article, indicate the article’s retracted status in the References list and also include a citation and full reference for the retraction notice.

🡪 We checked the reference list and corrected. No. 27 was missed and added. Number errors (27 -> 28, 28 -> 29) were corrected and No 29 was deleted

2. Abstract/Line 111: Spell out Randomized Controlled Trial before acronym of "RCT"

🡪 The ‘RCT’ was deleted from the abstract and presented as 'Randomized Controlled Trial'.

3. Lines 201-203: The new Table 2 including racial statistics includes six studies which authors state did not mention race, and they use the term "Nor." Further author investigation of those six studies as to participant race and explanation of "Nor" is requested.

-> ‘Nor reported’ was corrected to ‘Not reported.’ Six studies that did not mention race were reviewed again and the selected subject characteristics were added.

4. Line 420: With author manuscript revisions, the search timeline was expanded from ten to twelve years. Please update.

🡪 I updated search timeline from 10 years to 12 years.
